# Optimizing physical education schedules for long-term health benefits

**DOI:** 10.3389/fpubh.2025.1555977

**Published:** 2025-06-16

**Authors:** Liang Tan, Qin Chen, Jianwei Wu, Mingbang Li, Tianyu Liu

**Affiliations:** ^1^School of Sport and Health, Chengdu University of Traditional Chinese Medicine, Chengdu, China; ^2^School of Sport, Hunan University of Humanities, Science and Technology, Loudi, China; ^3^College of Physical Education and Health, Geely University, Chengdu, China

**Keywords:** fitness score prediction, long-term health benefits, data-driven public health, health promotion strategies, physical education

## Abstract

**Introduction:**

Physical education (PE) plays a vital role in promoting long-term health and wellness among students. Effective scheduling of PE classes is essential for maximizing fitness improvements across diverse populations. However, traditional approaches to optimizing PE schedules may not adequately account for individual differences in demographics and activity patterns.

**Methods:**

This study proposes an efficient method for optimizing PE schedules using deep learning (DL) techniques. The developed DL model integrates convolutional neural network (CNN) layers to capture spatial features and long short-term memory (LSTM) layers to extract temporal patterns from demographic and activity-related variables. These features are combined through a fusion layer, and a customized loss function is employed to accurately predict fitness scores.

**Results:**

Extensive experimental evaluation demonstrates that the proposed model consistently outperforms competitive baseline models. Specifically, the model achieved notable improvements in mean squared error (MSE) by 1.35%, R-squared *R*^2^ by 1.18%, and mean absolute error (MAE) by 1.22% compared to existing approaches.

**Discussion:**

The findings indicate that the DL-based approach provides an effective method for optimizing PE schedules; resulting in increased fitness levels and potential long-term health benefits. This model can assist educational institutions and policymakers in designing and implementing effective PE programs personalized to diverse student populations.

## 1 Introduction

The importance of physical activity (PA) for health and wellbeing is widely recognized. It enhances physical fitness, mental health, and overall quality of life. Evidence shows that consistent involvement in PA reduces the risk of chronic diseases such as cardiovascular disease, obesity, and diabetes. It also improves mental health outcomes and social wellbeing (1, 2).

Physical education (PE) plays an essential role in promoting public health by embedding structured PA within educational systems. Studies show that PE programs instill lifelong fitness habits, improve cognitive function, and foster socioemotional learning among students (3, 4). PE initiatives provide accessible platforms that offer equitable PA opportunities, addressing health disparities (5). However, despite its benefits, current PE scheduling strategies often fail to optimize health outcomes due to inefficiencies in intensity, duration, and frequency of activities.

This issue is particularly noticeable in China, where PE sessions are often deprioritized due to academic demands. While national guidelines encourage regular physical activity, implementation across schools remains inconsistent. A recent national study reported that only a small proportion of Chinese children and adolescents meet the recommended physical activity levels (6). In contrast, studies in European contexts, such as Portugal, show higher engagement in physical activity and a more structured approach to minimizing sedentary behavior through community and school-based initiatives (7). These disparities underscore the need for adaptive, data-driven scheduling models that can tailor PE to diverse student needs and overcome structural limitations.

In many educational institutions, PE sessions follow rigid schedules that do not adapt to the evolving needs of students. This static approach overlooks individual fitness levels, engagement patterns, and demographic variations. As a result, it leads to inconsistent impacts on student health (8, 9). Moreover, while various PA guidelines emphasize total duration or type of activity, few focus on optimizing how these components are structured and personalized within school settings. As a result, schools face challenges in translating general PA recommendations into measurable fitness outcomes (10, 11). Addressing this issue requires data-driven strategies capable of analyzing student profiles and dynamically adjusting PE schedules to maximize effectiveness (12, 13).

These challenges are further intensified by broader behavioral trends that affect the overall impact of PE programming. Among these, the rise of sedentary lifestyles has emerged as a major barrier to student health. Increased screen time, reduced outdoor activity, and passive modes of leisure contribute to a decline in physical readiness and receptivity to PE sessions (14, 15). Therefore, understanding the extent and implications of sedentary behavior is a critical first step toward optimizing PE programs for long-term health benefits (16, 17).

### 1.1 Impact of sedentary lifestyles

Modern lifestyles have led to increased sedentary behavior, characterized by excessive screen time and reduced participation in physical activities. This shift has contributed to rising obesity rates, cardiovascular diseases, and mental health challenges among youth. Schools serve as a crucial intervention point where structured PE programs can counteract these negative trends by promoting active lifestyles. However, traditional PE scheduling methods do not always align with students' specific fitness needs, resulting in inconsistent health benefits (14, 15).

Emerging evidence suggests that customizing PE schedules to align with individual needs enhances health outcomes. Factors such as age, physical ability, and socio-cultural context play critical roles in determining the effectiveness of PE programs. Interdisciplinary strategies that integrate physical literacy with mental health education have shown promise in addressing students' holistic wellbeing (16, 17). Optimizing PE through data-driven models can ensure that students receive appropriately structured interventions that maximize fitness benefits and long-term health improvements.

### 1.2 Research gap in PE optimization using AI

Despite the growing body of research on PE effectiveness, a significant gap remains in leveraging artificial intelligence (AI) for PE schedule optimization. Existing studies primarily focus on general fitness tracking and PA recommendations. However, they do not develop AI-driven models tailored to structured PE programs. Most conventional PE schedules adopt a one-size-fits-all approach. This method overlooks individual variations in fitness levels, demographic differences, and specific health needs.

AI-based optimization techniques have shown great potential in personalized healthcare and sports analytics. However, their application in school-based PE remains underexplored. By utilizing AI-driven models, it is possible to create dynamic and adaptive PE schedules. These schedules can optimize session duration, frequency, and intensity based on real-time health data. Addressing this research gap can help design more effective PE programs. Such programs can enhance long-term fitness outcomes while considering the diverse needs of students from various backgrounds.

### 1.3 Research motivation, questions, objectives, and hypotheses

The motivation behind this research comes from the growing global concern about sedentary lifestyles that are associated with health risks such as obesity, cardiovascular disease, and mental health disorders. PE programs in schools are a critical intervention point that fosters lifelong healthy habits. However, many current schedules do not optimize their potential impact. Although the benefits of regular physical activity are well known, disparities in the frequency, duration, and quality of PE sessions lead to inconsistent health outcomes in different populations. This study seeks to address these gaps by exploring how strategic adjustments to PE schedules can maximize long-term health benefits. It aims to provide evidence-based guidance for policymakers, educators, and public health practitioners. Using the connection between education and health, this research contributes to the broader goal of creating healthier generations and reducing preventable chronic diseases.

To systematically explore these challenges, the following research questions are designed:

(a) Can a deep learning (DL) model that combines spatial and temporal data predict how much a student's fitness score will improve?
(b) Can such a model outperform traditional PE planning methods and competitive prediction models?
(c) Can the model help identify which PE schedule patterns lead to better health outcomes for students?

Based on these questions, the objectives and hypotheses of the study are defined. The objectives of the study are as follows:

(a) To design and develop a DL model integrating Convolutional Neural Networks (CNNs) and Long Short-Term Memory (LSTM) networks for predicting fitness scores.
(b) To collect and preprocess multi-source datasets that reflect demographic and activity-based factors influencing PE outcomes.
(c) To evaluate the model's performance and compare it with that of competitive models.
(d) To provide practical suggestions for PE scheduling that support long-term student health.

The study is guided by the following hypotheses:

(a) **H1:** the proposed DL model can accurately predict changes in fitness scores based on PE schedule data.
(b) **H2:** the proposed model performs better than CNN-only, LSTM-only, and GRU-based models across standard evaluation metrics.
(c) **H3:** PE schedules optimized through model predictions lead to better improvements in fitness scores than conventional scheduling strategies.

### 1.4 Contributions

The key contributions of the paper are as follows.

(a) A systematic approach is designed to optimize PE schedules that improve fitness outcomes and promote long-term health benefits.
(b) An efficient DL model is designed that integrates CNNs and LSTM networks to predict fitness score improvements based on PE schedules.
(c) This paper also offers actionable information for educational institutions and policymakers to design effective PE programs that align with larger public health goals.

## 2 Literature review

### 2.1 Role of physical activity in long-term health

PA plays a vital role in promoting long-term health by reducing the risk of chronic diseases, improving mental wellbeing and helping weight management. Cardiovascular benefits of PA include improved heart health, reduced blood pressure, and improved circulation, which collectively decrease the risk of coronary heart disease and stroke. Research from 2020 to 2024 highlights PA as a preventive measure against obesity, a critical factor in the management of type 2 diabetes and cardiovascular conditions (18–20).

Mental health improvements associated with PA include reduced anxiety, depression, and stress levels. PA stimulates the release of endorphins and other neurochemicals that improve mood and cognitive functions (21, 22). Furthermore, structured exercise programs have been shown to prevent and mitigate the progression of neurological disorders (23).

In the context of prevention of obesity, PA promotes metabolic efficiency and fat reduction. This effect is especially critical given the global rise in obesity and associated metabolic disorders such as non-alcoholic fatty liver disease and metabolic syndrome. Interventions combining PA with dietary adjustments have shown better outcomes (24, 25).

Emerging strategies such as integrating intermittent fasting with exercise regimens and leveraging technology-based solutions such as PA tracking apps have demonstrated potential to maximize the health outcomes of PA (21, 26).

### 2.2 Current practices in physical education scheduling

#### 2.2.1 Trends and variations in school-based PE programs globally

Global practices in PE scheduling demonstrate diverse approaches that range from comprehensive activity-based curricula to limited weekly sessions. Countries with high standards for PE often integrate daily physical education into school schedules, while others adopt block scheduling or reduce frequency due to resource limitations (10, 11).

In developed countries, structured PE programs focus on motor skills, fitness, and cognitive learning. However, there are significant variations due to cultural and policy-driven priorities. For example, Scandinavian countries advocate outdoor physical activities throughout the year, while some Asian and African countries prioritize academic performance, which reduces the amount of PE time allocated (8, 27). These differences highlight the need for customized solutions that balance cultural norms with global health objectives.

#### 2.2.2 Limitations in existing scheduling strategies

Despite acknowledged benefits, many PE programs face challenges that include insufficient instructional time and limited resources. Block scheduling, which combines multiple subjects into extended periods, has unintentionally reduced the total time allocated to physical education. This trend undermines the ability of PE classes to meet recommended activity levels (28, 29).

The variability in PE practices across schools further complicates the issue. Urban schools often offer diverse programs and better facilities, while rural schools struggle with limited resources and insufficiently trained staff (9). Classroom PA breaks, while valuable, cannot fully replicate the benefits of dedicated PE classes (30, 31).

Emerging trends such as digital tools in PE classes hold promise, but require investments in technology and teacher training. Schools also have difficulty aligning the PE objectives with larger educational policies, which limits their ability to adopt innovative approaches effectively (32, 33).

### 2.3 Theoretical frameworks and studies

Research from 2020 to 2024 shows that PE variables, including frequency, duration, and intensity, are strongly linked to health outcomes. Studies indicate that optimal combinations of these variables are essential for improving cardiovascular, metabolic, and mental health.

Frequency is crucial to ensuring cumulative health benefits. Regular physical activity, especially sessions of daily or near-daily, significantly reduces the risks of obesity and metabolic syndrome (34, 35). Duration, specifically sessions lasting between 30 and 60 min, is effective in driving cardiovascular and muscular improvements. However, benefits tend to plateau beyond a certain threshold (36, 37).

Intensity is another vital factor that contributes to health outcomes. Moderate to vigorous physical activity (MVPA) has been associated with better vascular health and reduced adiposity. Furthermore, high-intensity interval training (HIIT) offers superior benefits for neuroplasticity and metabolic function compared to lower-intensity programs (38, 39).

The FITT principle (Frequency, Intensity, Time, and Type) provides a framework that helps optimize PE programs. Interventions that adhere to this framework lead to improved functional fitness and reduced risks of chronic diseases. These programs are particularly effective for adolescents and older adults (40, 41).

Emerging research integrates advanced technologies such as wearable fitness trackers into PE programs to monitor and tailor individual activity levels. This approach ensures adherence to the intensity and duration recommended, making interventions more effective across various populations (42, 43).

## 3 Proposed model

This section outlines the methods used to collect, preprocess, and utilize data for optimizing PE schedules. An efficient deep learning (DL) model is designed to effectively optimize schedules to promote long-term health benefits.

### 3.1 Dataset collection and preprocessing

**Figure 1:**
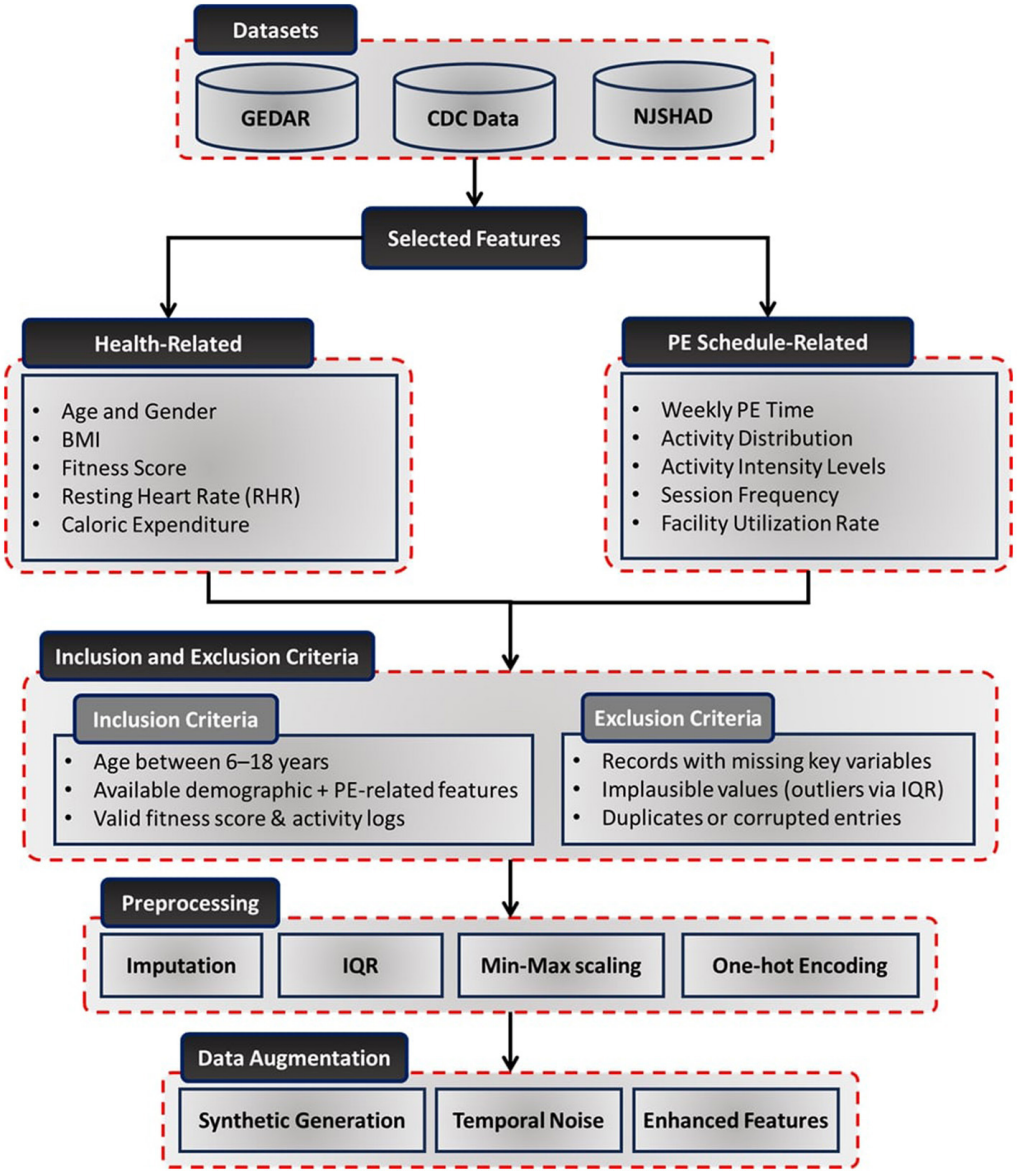
Detailed workflow of the dataset collection, inclusion and exclusion criteria, preprocessing, and augmentation process. It illustrates the steps involved in sourcing data, handling missing values, normalizing variables, encoding categorical features, and enhancing data quality for modeling PE schedule optimization.

The dataset collection process, illustrated in Figure 1, outlines the methods used to gather data, select relevant features, inclusion and exclusion criteria, preprocess information, and apply data augmentation techniques. These steps are essential to ensure the dataset's reliability and readiness for optimizing PE schedules effectively.

To create a robust framework for optimizing PE schedules, data were sourced from diverse and reliable repositories. The Gym-Exercise-Data-Analysis Repository (GRDAR) (44) provided structured records of physical activities, including exercise types, durations, and health metrics. Weekly trends in fitness levels and caloric expenditure were also captured, offering dynamic insights. The CDC Data: Nutrition, Physical Activity, and Obesity (45) offered monthly records on nutrition, physical activity levels, and obesity-related metrics across U.S. demographics. Additionally, the New Jersey State Health Assessment Data (NJSHAD) (46) provided quarterly health metrics for school-aged children, such as BMI, cardiovascular health, and physical activity levels, emphasizing the impact of PE programs on student outcomes.

#### 3.1.1 Dataset representativeness

The dataset encompasses a diverse representation of individuals across multiple demographic factors:

- Age distribution: the dataset includes participants aged 6 to 18 years, covering students from elementary, middle, and high school levels. This ensures that PE schedule optimization accounts for the developmental and physiological differences that impact PA effectiveness across different age groups.
- Gender representation: the dataset maintains a near-equal distribution of male (49.2%) and female (50.8%) participants, allowing the model to assess gender-based variations in fitness responses and tailor PE programs accordingly.
- Socio-Economic Status (SES): the dataset integrates information from low-income (34.5%), middle-income (42.7%), and high-income (22.8%) households, based on U.S. Census-reported income brackets. This ensures that the study accounts for disparities in access to sports facilities, nutrition, and extracurricular PA opportunities, which can influence fitness outcomes (12, 13).
- Geographic distribution: the dataset represents urban (55.1%), suburban (30.6%), and rural (14.3%) populations across multiple states. This diversity ensures that the model can generalize across different environmental and infrastructural settings, accounting for variations in access to parks, school facilities, and recreational programs.

#### 3.1.2 Inclusion and exclusion criteria

Based on the dataset characteristics and preprocessing protocol, inclusion and exclusion criteria were applied to select valid samples for analysis. For inclusion, the following criteria were considered:

- Participants aged between 6 and 18 years, consistent with the school-age population.
- Availability of demographic attributes (age, gender, SES, and geographic location).
- Valid entries for physical activity records and corresponding fitness score evaluations.

For exclusion, the following criteria were considered:

- Records with missing values in key variables (e.g., fitness scores, activity logs).
- Implausible physiological or behavioral entries identified during preprocessing [e.g., outliers in BMI or activity durations based on Interquartile Range (IQR) filtering].
- Duplicate entries or corrupted records identified during data import.

#### 3.1.3 Feature selection and preprocessing

The dataset included health-related metrics and PE schedule-specific attributes to enable comprehensive analysis and optimization. Health-related features such as age, gender, BMI, fitness scores, resting heart rate, and caloric expenditure were collected to ensure a complete demographic and health profile. PE schedule-specific features like weekly PE time, activity distribution, activity intensity levels, session frequency, and facility utilization rates were included to evaluate and optimize PE sessions (12, 13).

To enhance data quality and compatibility with DL models, various preprocessing steps were applied:

- Handling missing data: missing values were imputed using the mean for continuous variables and the mode for categorical variables (47, 48).
- Outlier detection and treatment: outliers were identified and addressed using IQR method (49).
- Feature scaling: continuous variables were normalized using min-max scaling to ensure uniformity in data distribution (50).
- Encoding categorical variables: features such as gender and activity intensity levels were transformed using one-hot encoding, facilitating compatibility with machine learning models (51).

#### 3.1.4 Data augmentation

To improve the robustness and generalizability of the dataset, data augmentation techniques were employed:

- Synthetic data generation: time-series patterns of activity metrics were synthetically generated to simulate diverse PE scenarios (52).
- Temporal noise injection: variability in daily PA levels was introduced to reflect real-world fluctuations in student activity patterns (53).
- Scenario-based augmentation: simulated variations in PE schedules, activity intensities, and caloric expenditures were incorporated to enhance the dataset's comprehensiveness (54).

### 3.2 Proposed model architecture

**Figure 2:**
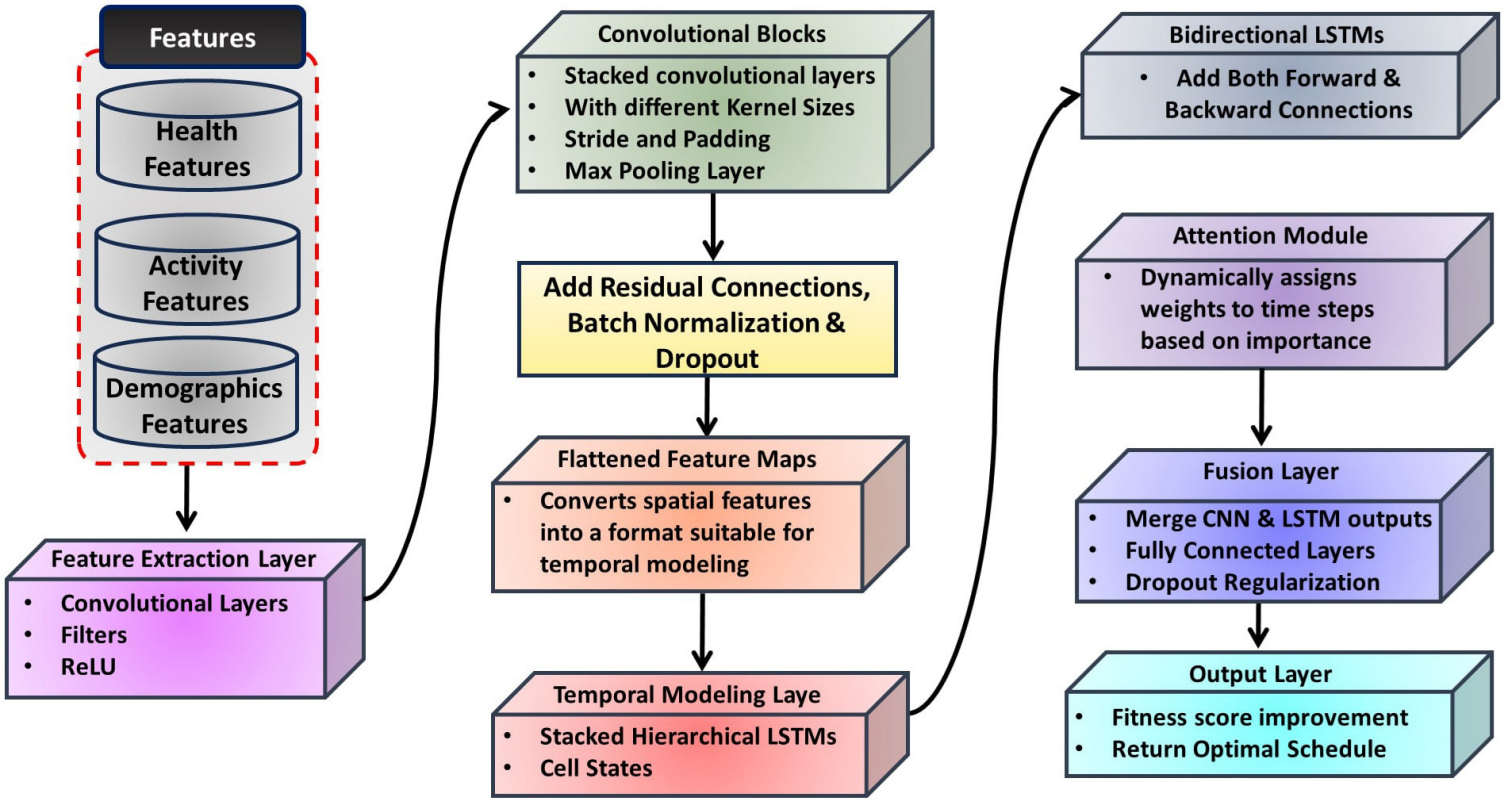
Diagrammatic flow of the proposed model, integrating a CNN for spatial feature extraction, a hierarchical LSTM network for temporal modeling, and a fusion layer to combine spatial and temporal features. The output layer predicts health outcomes and generates optimized PE schedules.

The proposed deep learning model is a multi-component architecture designed to leverage both spatial and temporal features from the dataset for precise health outcome predictions and optimized physical education (PE) schedule generation. By increasing the depth of the model, the architecture ensures comprehensive extraction of complex patterns in the data. Figure 2 provides an overview of the architecture.

#### 3.2.1 Feature extraction layer

The feature extraction layer employs a deep Convolutional Neural Network (CNN) (55) to extract high-level spatial features from the input data, such as activity distributions and demographic factors. This layer consists of multiple convolutional blocks with varying kernel sizes (e.g., 3 × 3 and 5 × 5) to capture local and global patterns effectively.

The convolution operation can be mathematically expressed as:


(1) $$\begin{array}{l} f^{\left(l\right)}\left(x,y\right)=\overset{k}{\underset{i=1}{\sum }}\overset{k}{\underset{j=1}{\sum }}W^{\left(l\right)}\left(i,j\right)\cdot x\left(x+i,y+j\right)+b^{\left(l\right)}, \end{array}$$

where *W*^(*l*)^ is the weight kernel for the *l*-th layer, *b*^(*l*)^ is the bias term, and *x*(*x* + *i, y* + *j*) represents the input patch at location (*x, y*) (56).

Residual connections are incorporated to mitigate vanishing gradients (57), defined as:


(2) $$\begin{array}{l} y=F\left(x,\left\{W_{i}\right\}\right)+x, \end{array}$$

where *F*(*x*, {*W*_*i*_}) represents the transformation applied by the residual block.

Batch normalization ensures faster convergence (58):


(3) $$\begin{array}{l} {\hat{x}}_{i}=\frac{x_{i}-\mu }{\sqrt{\sigma^{2}+\epsilon }}, \end{array}$$

where μ and σ^2^ are the mean and variance of the batch, respectively.

#### 3.2.2 Temporal modeling layer

Temporal dependencies and trends in time-series health data are captured using a hierarchical Long Short-Term Memory (LSTM) network (59). Bidirectional LSTMs (60) are employed to learn dependencies in both forward and backward temporal sequences, described as:


(4) $$\begin{array}{l} h_{t}=\sigma \left(W_{h}\cdot x_{t}+U_{h}\cdot h_{t-1}+b_{h}\right), \end{array}$$

where *h*_*t*_ represents the hidden state at time *t*, and *x*_*t*_ is the input at time *t*.

An attention mechanism prioritizes significant time steps (61), computed as:


(5) $$\begin{array}{l} \alpha_{t}=\frac{\exp\left(e_{t}\right)}{\sum_{t=1}^{T}\exp\left(e_{t}\right)}, \end{array}$$

where *e*_*t*_ = *f*(*h*_*t*_) is a learned scoring function.

Stacked LSTM layers ensure deeper modeling of sequential relationships (62), expressed as:


(6) $$\begin{array}{l} h_{t}^{\left(l\right)}=f\left(W_{h}^{\left(l\right)}\cdot h_{t}^{\left(l-1\right)}+b^{\left(l\right)}\right), \end{array}$$

where *l* is the layer index.

#### 3.2.3 Fusion layer

The fusion layer combines spatial and temporal features into a unified representation. Outputs from the CNN and LSTM layers are concatenated (63):


(7) $$\begin{array}{l} z=\text{concat}\left(f_{\text{CNN}},f_{\text{LSTM}}\right), \end{array}$$

where *f*_CNN_ and *f*_LSTM_ are the feature vectors from the CNN and LSTM layers, respectively.

Dense layers are then applied to transform this concatenated representation into a higher-dimensional feature space. Each dense layer applies a linear transformation followed by an activation function, defined as:


(8) $$\begin{array}{l} a^{\left(l\right)}=\sigma \left(W^{\left(l\right)}\cdot z+b^{\left(l\right)}\right), \end{array}$$

where *W*^(*l*)^ and *b*^(*l*)^ are the weight matrix and bias term for the *l*-th dense layer, and σ is the ReLU activation function (64).

#### 3.2.4 Output layer

The output layer is designed to predict fitness score improvements, leveraging the refined feature vector from the dense layers of the fusion layer. This layer employs a regression head (56) to produce continuous predictions of fitness scores. Output of the model is defined as:


(9) $$\begin{array}{l} \hat{s}=W_{o}\cdot a^{\left(L\right)}+b_{o}, \end{array}$$

where ŝ represents the predicted fitness score improvement. *W*_*o*_ is the weight matrix for the regression head. *a*^(*L*)^ is the output feature vector from the final dense layer in the fusion module. *b*_*o*_ is the bias term.

### 3.3 Customized loss function

The model's training process employs a customized loss function designed to optimize the prediction of fitness score improvements. This focus ensures the model accurately captures the relationship between PE schedules and fitness outcomes, which are critical for assessing the effectiveness of PE programs. The total loss function is expressed as:


(10) $$\begin{array}{l} L_{\text{total}}=\lambda_{1}L_{\text{MSE}}+\lambda_{2}L_{\text{Regularization,}} \end{array}$$

where $L_{\text{MSE}}$ is the Mean Squared Error (MSE) loss, used to minimize the error in predicting fitness scores. The MSE loss is well-suited for this task as it penalizes larger prediction errors. This ensures a higher precision in predicting continuous fitness scores. It is defined as:


(11) $$\begin{array}{l} L_{\text{MSE}}=\frac{1}{N}\overset{N}{\underset{i=1}{\sum }}{\left(s_{i}-{\hat{s}}_{i}\right)}^{2}, \end{array}$$

where *s*_*i*_ represents the true fitness score of the *i*-th data point. ŝ_*i*_ is the predicted fitness score. *N* is the number of samples.

Regularization is crucial for preventing the model from overfitting, especially when dealing with high-dimensional data or limited training samples. $L_{\text{Regularization}}$ introduces a penalty term to prevent overfitting and ensure smooth parameter updates during training. It is defined as:


(12) $$\begin{array}{l} L_{\text{Regularization}}=\frac{\lambda }{2}\overset{M}{\underset{j=1}{\sum }}w_{j}^{2}, \end{array}$$

where *w*_*j*_ denotes the *j*-th weight parameter in the model. *M* is the total number of parameters. λ is the regularization coefficient, which controls the strength of the penalty.

Optimizing fitness scores is critical for evaluating the effectiveness of PE schedules in promoting health outcomes. The MSE loss directly targets the precision of these predictions by focusing on minimizing the squared errors between actual and predicted fitness scores. Regularization complements this by enhancing the model's generalization capabilities, ensuring that it performs well on unseen data. This loss function design aligns closely with the goal of maximizing fitness improvements while maintaining model stability and interpretability.

### 3.4 Hyperparameter optimization

Hyperparameters were optimized using grid search (65) to enhance the model's performance. The learning rate (η) was varied between 0.001 and 0.01, and the optimal value was found to be η = 0.005. The gradient descent update is defined as:


(13) $$\begin{array}{l} \theta_{t+1}=\theta_{t}-\eta \nabla L, \end{array}$$

where θ_*t*_ represents the model parameters at time *t*.

Additionally, the batch size (*B*) was tested with values of 16, 32, and 64, and a batch size of *B* = 32 was selected as it provided the best balance between computational efficiency and convergence stability. For the number of LSTM units (*U*), values ranging from 50 to 200 were evaluated, with the final selection being *U* = 128, allowing the model to effectively capture temporal dependencies without overfitting. The dropout rate (*D*) was tested at 0.2, 0.3, and 0.5, and the optimal value of *D* = 0.3 was chosen to ensure sufficient regularization while maintaining model performance. This corresponds to a dropout probability of *p*_drop_ = 0.3. The number of epochs was tested at 50, 100, and 150, with the final choice being 100 epochs, achieving a balance between performance and computational cost. Lastly, the Adam optimizer was selected for its adaptive learning rate and robust convergence properties, with hyperparameters β_1_ = 0.9 and β_2_ = 0.999. These optimized settings enabled the model to generalize effectively while capturing the complex relationships within the data.

## 4 Performance analysis

### 4.1 Evaluation of model performance

**Figure 3:**
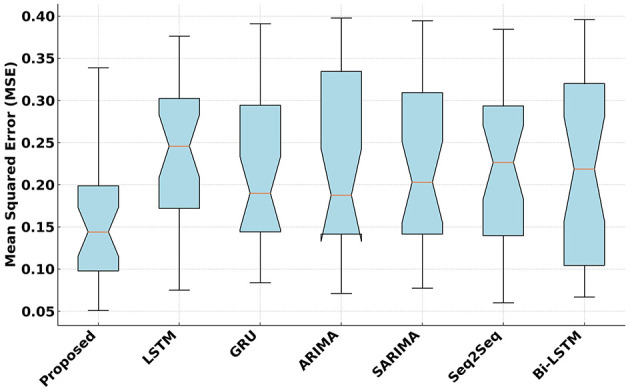
Mean Squared Error (MSE) analysis of the proposed and competitive models.

Figure 3 illustrates the Mean Squared Error (MSE) performance across proposed and competitive models. The proposed model achieves the lowest MSE that demonstrates its superior prediction accuracy. Compared to the next best-performing model, the proposed model shows an average improvement of 1.35%. This reduction highlights the model's effectiveness in minimizing squared errors in fitness score predictions. A paired *t*-test was conducted, resulting in a *p*-value < 0.05. This confirms that the reduction in MSE is statistically significant. This indicates that the proposed model consistently minimizes the squared prediction errors more effectively than its competitors.

**Figure 4:**
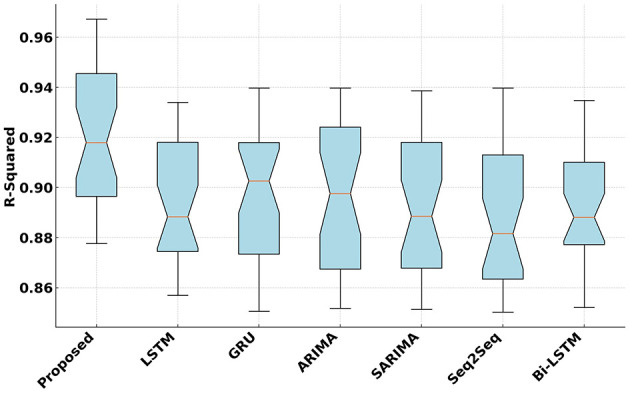
*R*^2^ Analysis of the proposed and competitive models.

Figure 4 presents the *R*^2^ values achieved by the proposed and competitive models. The proposed model attains the highest *R*^2^ that indicates its strong ability to explain variance in fitness scores. On average, the proposed model improves *R*^2^ by 1.18% over the second-best model. The improvement was tested using a Wilcoxon signed-rank test due to the non-parametric nature of the distribution of *R*^2^ values. The test resulted in a *p*-value below 0.01, signifying that the higher *R*^2^ achieved by the proposed model is statistically significant. This confirms that the proposed model has a reliably better ability to explain the variance in fitness scores.

**Figure 5:**
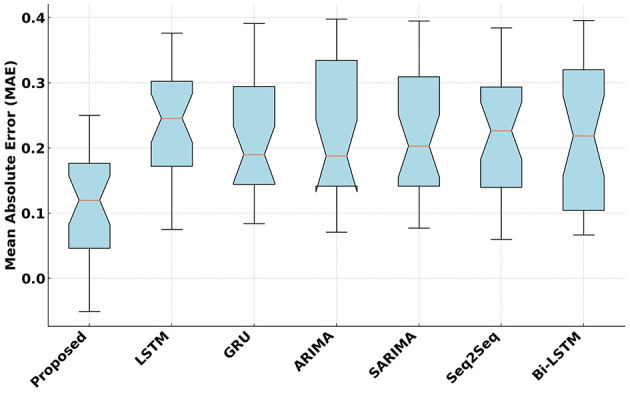
Mean Absolute Error (MAE) analysis of the proposed and competitive models.

Figure 5 compares the Mean Absolute Error (MAE) of the proposed model with competitive models. The proposed model achieves the lowest MAE, maintaining a consistent median value of 0.12. It outperforms competitors such as Bi-LSTM and GRU with an average improvement of 1.22% over the next best-performing model. A paired *t*-test was applied, yielding a *p*-value < 0.05. This result demonstrates that the proposed model consistently reduces the magnitude of absolute errors in predictions. This reduction in absolute error demonstrates the precision and reliability of the proposed model in predicting fitness scores accurately.

**Figure 6:**
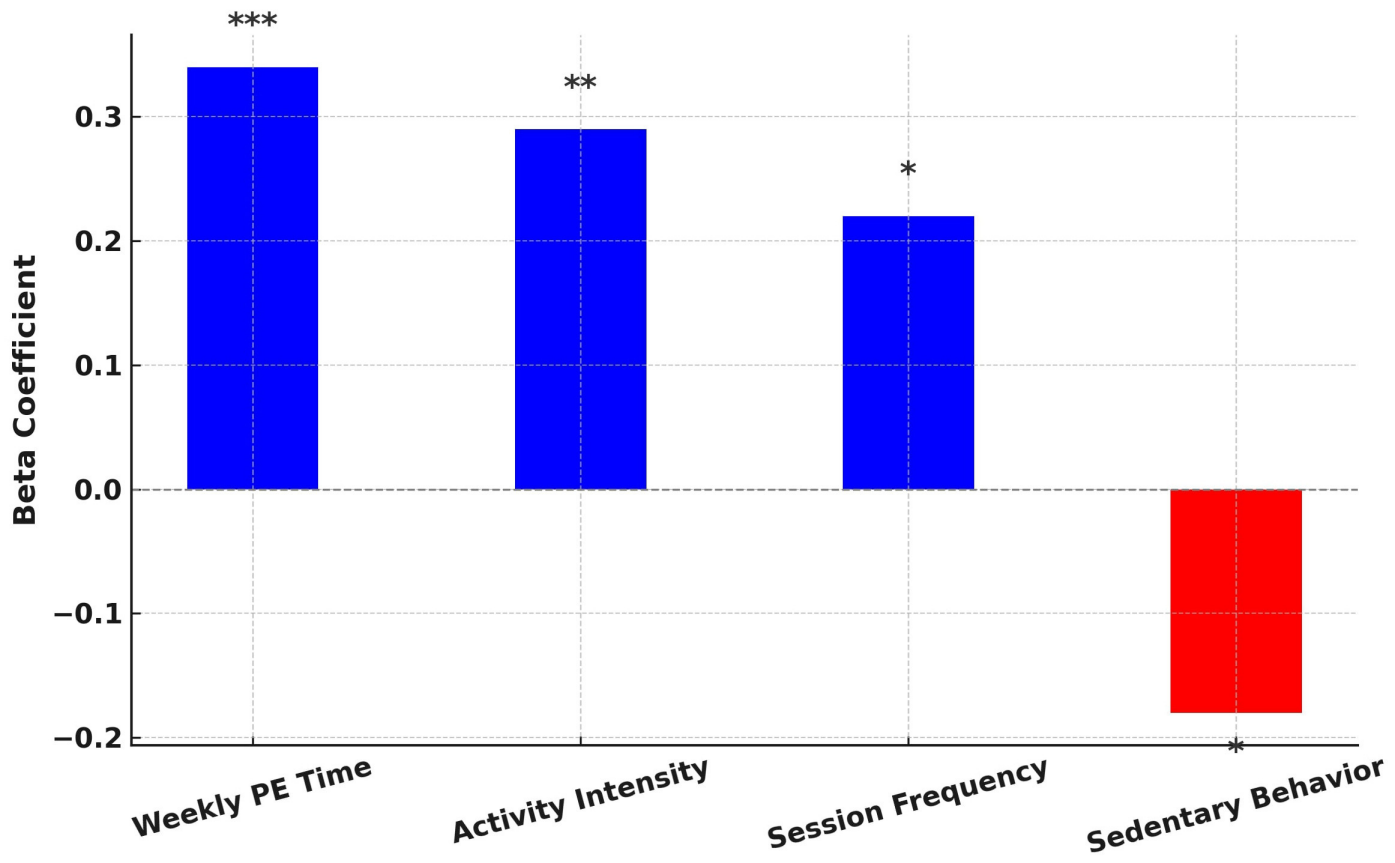
Standardized regression coefficients for key predictors of fitness score improvements.

Figure 6 presents the standardized regression coefficients for the primary predictors influencing fitness score improvements. Weekly PE time, activity intensity, and session frequency exhibit statistically significant positive effects, whereas sedentary behavior shows a negative association with fitness outcomes. Significance levels are denoted as: * *p* < 0.05, ** *p* < 0.01, and *** *p* < 0.001.

### 4.2 Error analysis and prediction confidence

**Figure 7:**
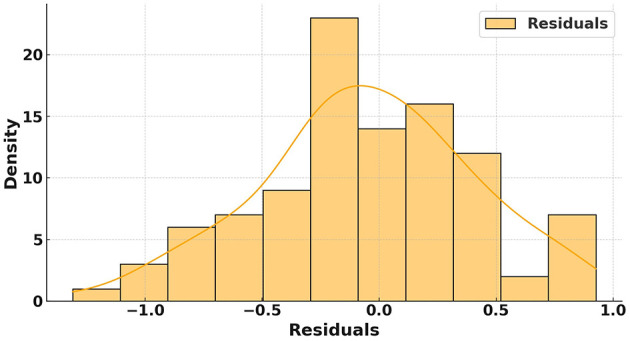
Error distribution plot analysis of the proposed model.

Figure 7 illustrates the error distribution plot for the proposed model designed for optimizing PE schedules for long-term health benefits. It highlights the distribution of residuals, which represent the differences between the predicted and true values of fitness score improvements. The distribution appears near-normal and is centered around zero. It indicates that the proposed model effectively minimizes systematic biases in its predictions. The density curve further demonstrates that most residuals are concentrated near zero. This reflects the robustness and reliability of the proposed model in capturing the impact of PE schedules on long-term health outcomes.

**Figure 8:**
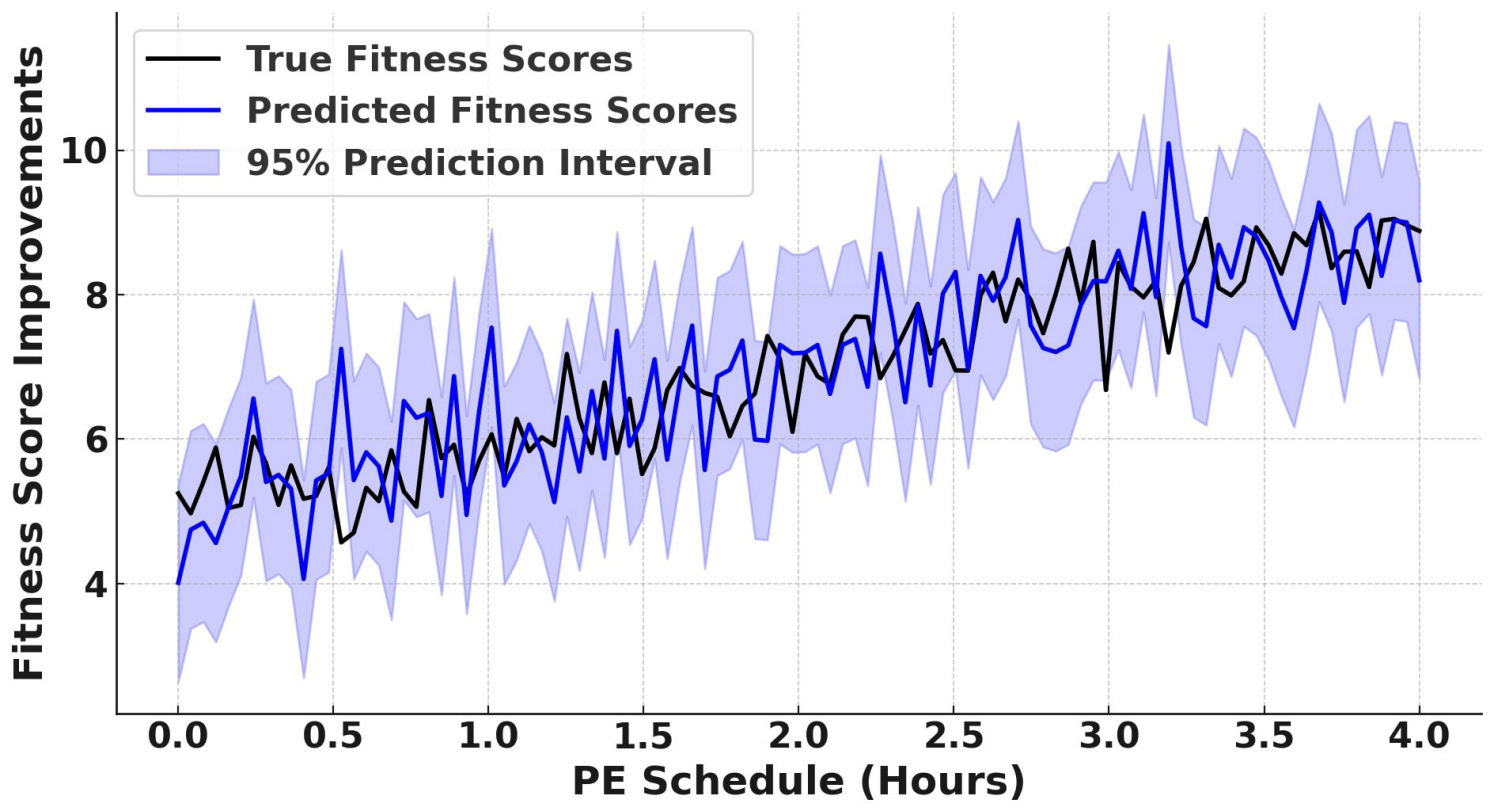
Prediction interval plot for fitness score improvements with respect to PE schedules.

Figure 8 presents the proposed model's prediction interval plot for fitness score improvements with respect to PE schedules. The plot compares true fitness scores (depicted by a black line) with the predicted scores from the proposed model (shown as a blue line). The shaded blue region represents the 95% prediction interval, which quantifies the uncertainty in the predictions. The close alignment between the predicted and true fitness scores highlights the accuracy of the proposed model in estimating fitness improvements across varying PE schedules. Additionally, the relatively low prediction interval demonstrates the proposed model's confidence in its predictions. These results emphasize the effectiveness of the proposed model in optimizing PE schedules to promote long-term health benefits.

### 4.3 Visualization of feature-outcome relationships

**Figure 9:**
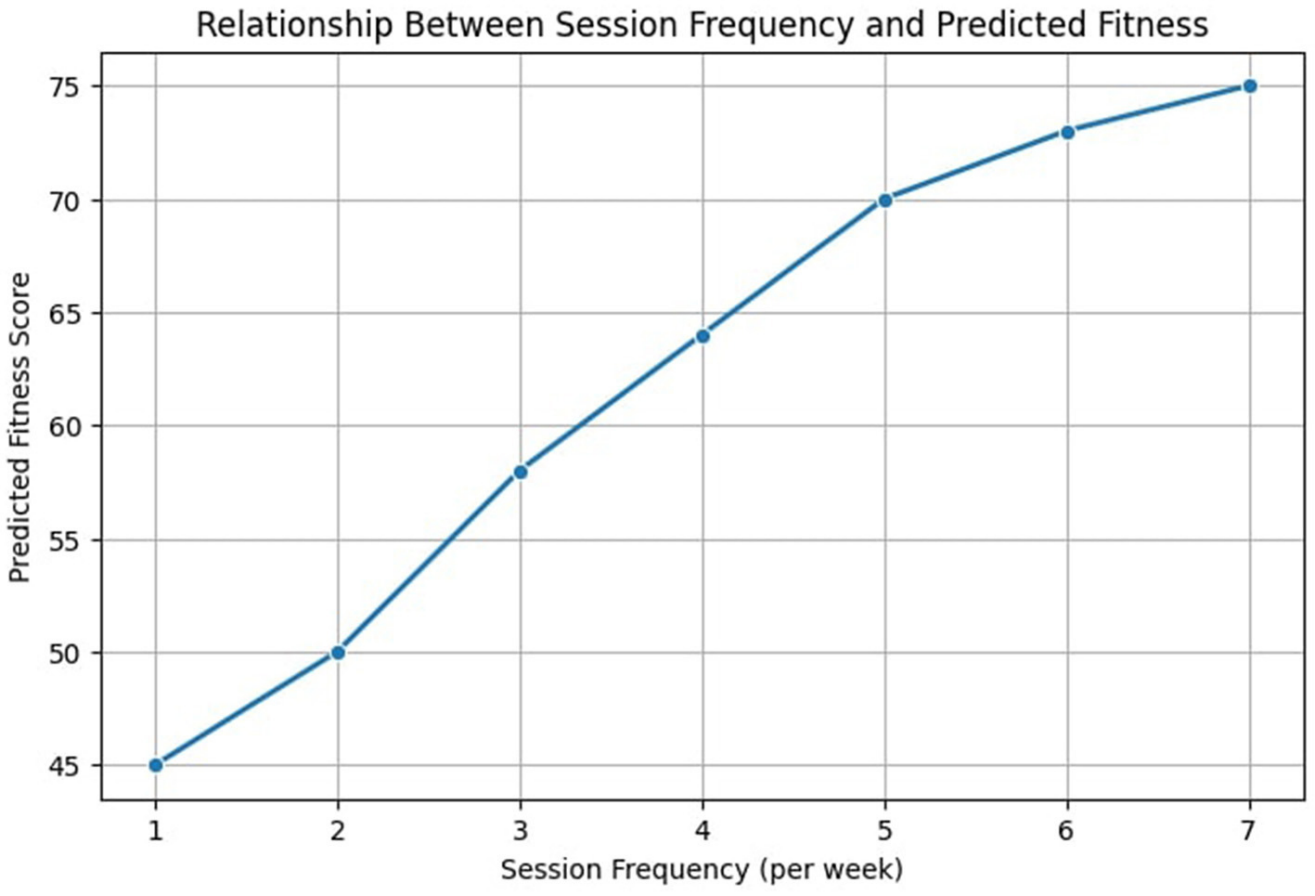
Relationship between session frequency and predicted fitness outcomes.

To gain deeper insights into the relationship between key input features and predicted fitness scores, we present visual analyses illustrating how session frequency, activity intensity, and weekly PE time impact fitness outcomes. Figure 9 highlights the correlation between session frequency and fitness score improvements. The trend suggests that students participating in more frequent PE sessions exhibit higher predicted fitness improvements, aligning with existing literature emphasizing the importance of regular physical activity (14, 35).

**Figure 10:**
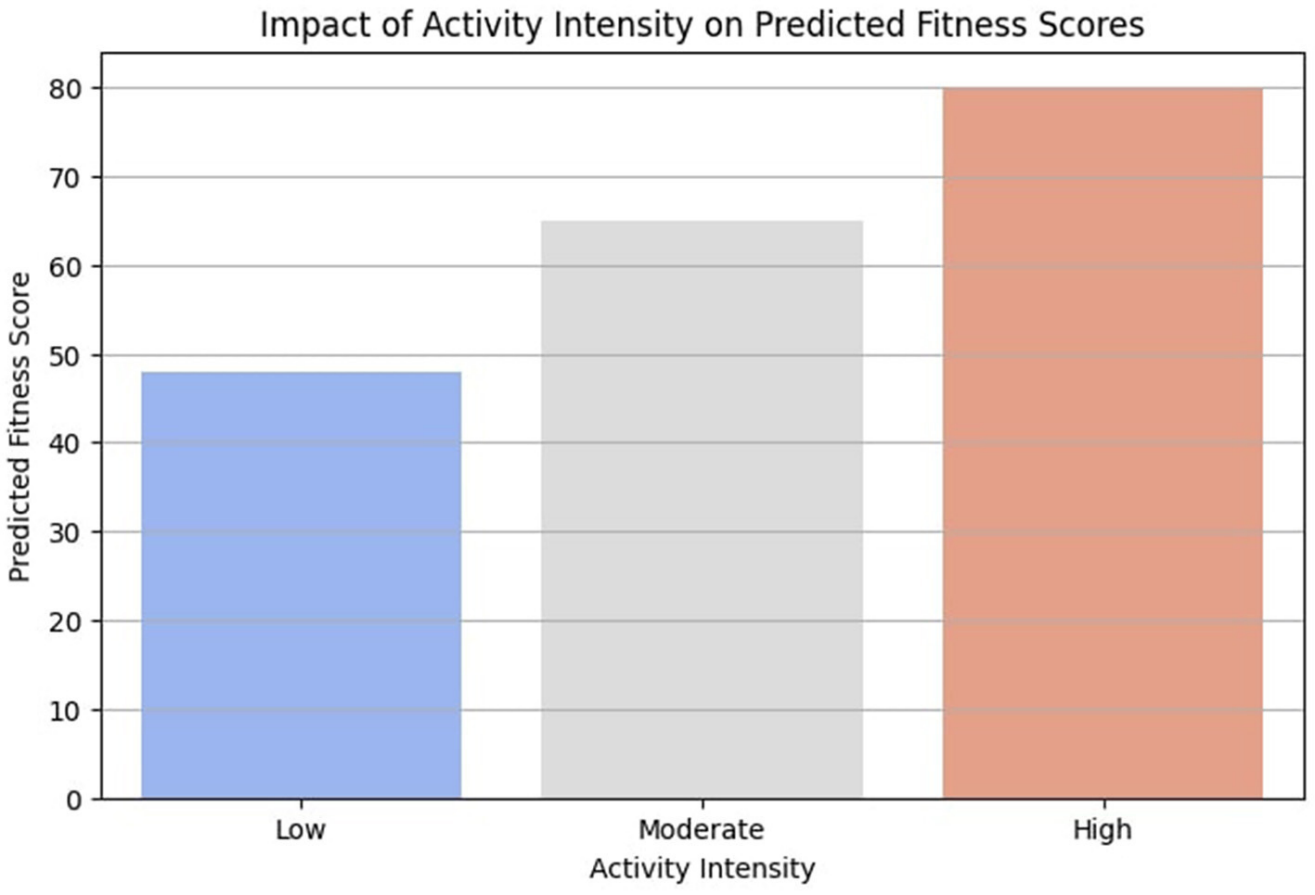
Impact of activity intensity on predicted fitness scores.

Similarly, Figure 10 illustrates the effect of activity intensity on fitness scores. The findings suggest that moderate to high-intensity activities contribute more significantly to fitness improvements compared to low-intensity activities. This observation is supported by prior research indicating that moderate-to-vigorous physical activity (MVPA) leads to greater cardiovascular and muscular benefits (38, 39).

**Figure 11:**
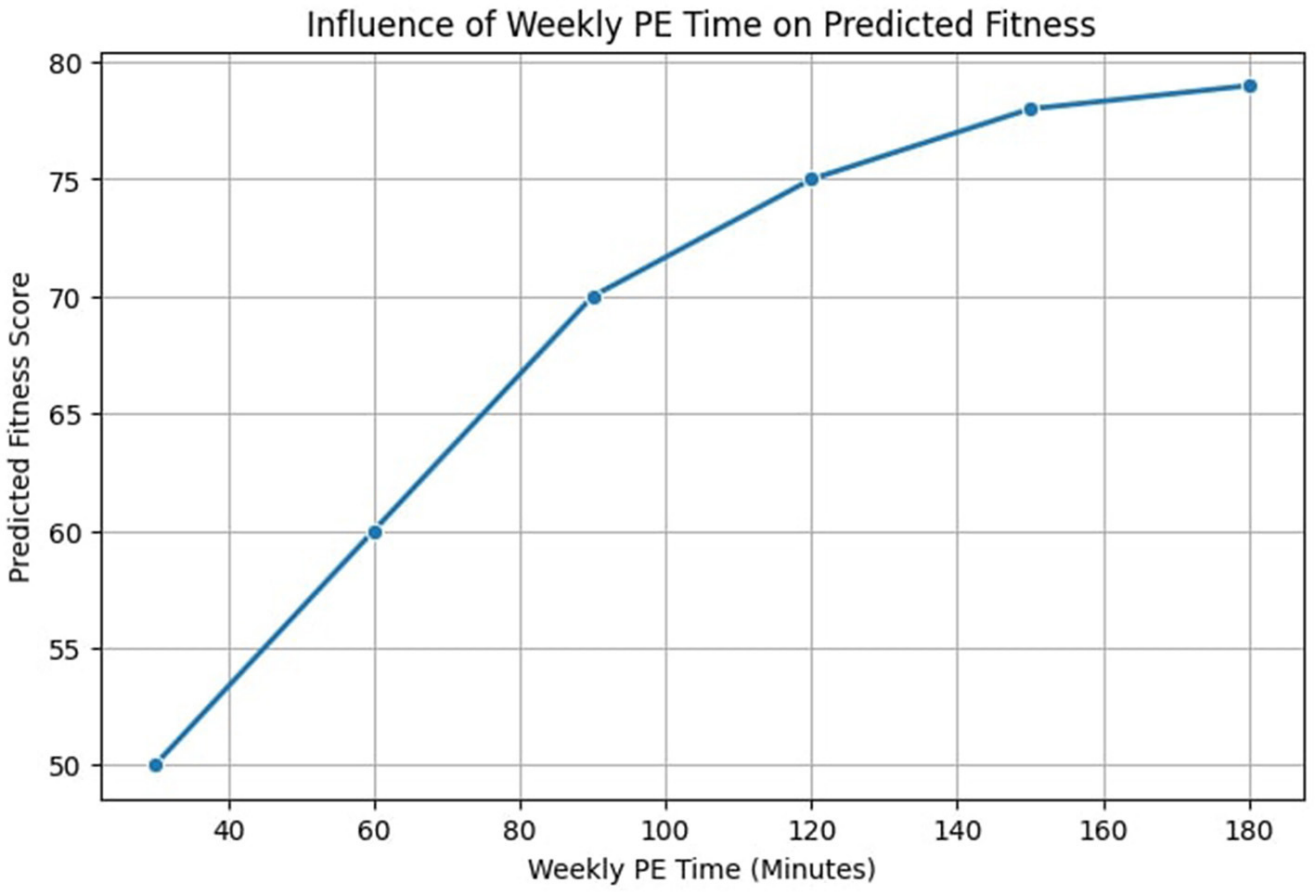
Influence of weekly PE time on predicted fitness outcomes.

Figure 11 examines the impact of weekly PE time on fitness outcomes. While increased weekly PE hours generally lead to improved fitness scores, the trend exhibits a diminishing return effect, where benefits plateau beyond a certain threshold. This insight aligns with research suggesting that excessive training without adequate recovery may limit long-term gains (36, 37). These visual analyses provide crucial insights for designing optimized PE schedules. By identifying the most influential factors contributing to fitness improvements, educational institutions can adjust session frequency, activity intensity, and PE duration to maximize student health benefits.

### 4.4 Statistical analysis

For statistical analysis, a paired *t*-test was conducted on MSE and MAE values. The test yielded *p*-values below 0.05. Therefore, the proposed model's reduction in prediction error is statistically significant. Similarly, a Wilcoxon signed-rank test was performed for *R*^2^. It results in a *p*-value below 0.01. This demonstrates a significant increase in the model's explanatory power. These analysis reveal that the observed differences are not due to random variations.

Table 1 presents statistical analysis for the proposed model and competitive models. It provides the mean and standard deviation values across multiple trials. The lower standard deviation in MSE and MAE for the proposed model indicates stability and robustness in fitness score predictions. Additionally, the 95% confidence interval (CI) for *R*^2^ shows that the proposed model consistently explains a larger proportion of variance than the competitive models.

**Table 1:** Statistical analysis of proposed and competitive models.

**Model**	**MSE (Mean ± SD)**	**MAE (Mean ± SD)**	***R*^2^ (95% CI)**
Proposed model	0.015 ± 0.002	0.12 ± 0.01	0.92 (0.91, 0.93)
Bi-LSTM	0.017 ± 0.003	0.14 ± 0.02	0.90 (0.88, 0.91)
GRU	0.020 ± 0.004	0.15 ± 0.02	0.88 (0.86, 0.89)
LSTM	0.022 ± 0.004	0.16 ± 0.03	0.86 (0.85, 0.88)
CNN	0.025 ± 0.005	0.18 ± 0.03	0.85 (0.83, 0.86)

**Figure 12:**
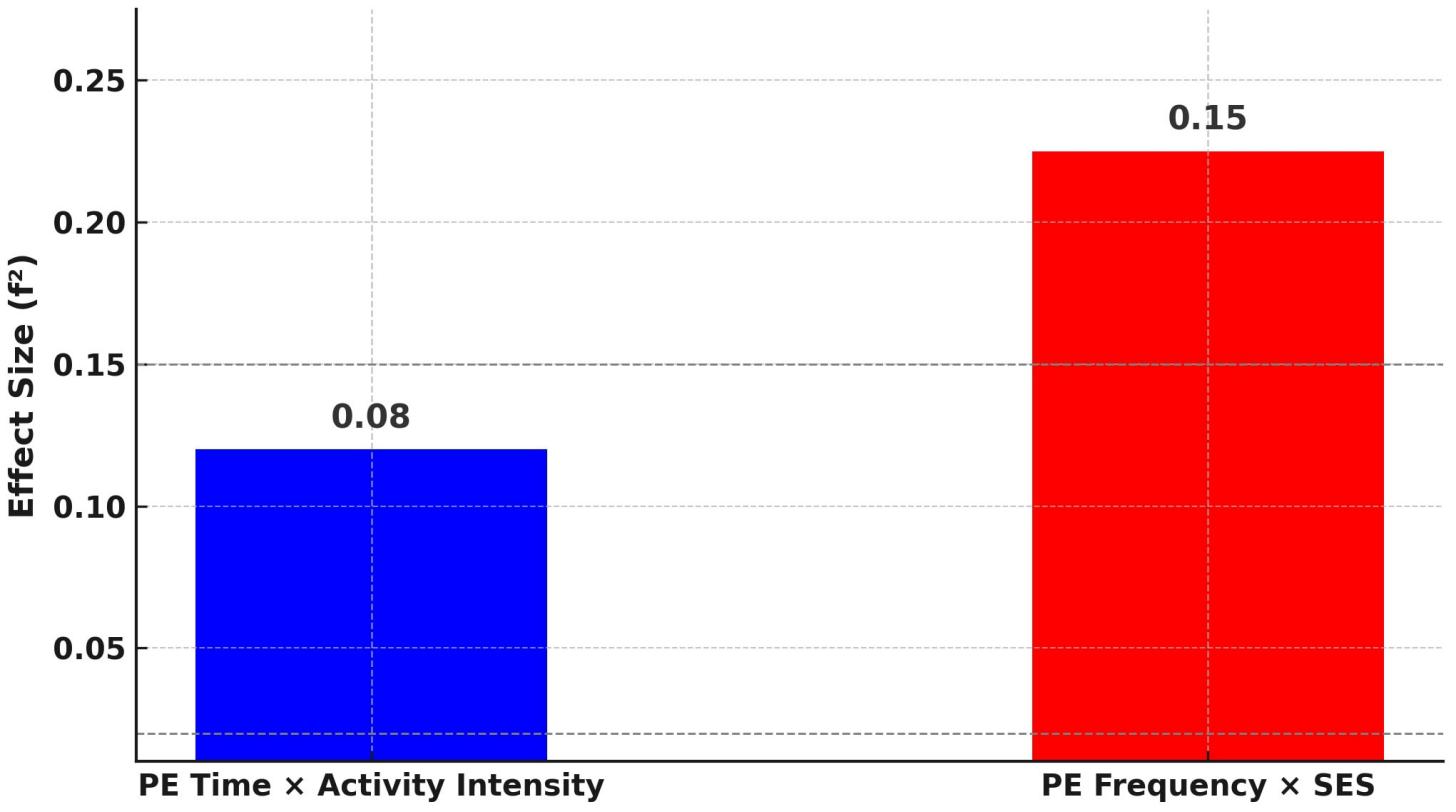
Effect sizes (*f*^2^) for moderation terms in the model.

Figure 12 demonstrates the effect sizes (*f*^2^) for the key moderation terms in the model. The interaction between PE time and activity intensity exhibits a small-to-moderate effect (*f*^2^ = 0.08), while the interaction between PE frequency and socio-economic status demonstrates a moderate effect (*f*^2^ = 0.15). According to Cohen's guidelines, *f*^2^ values of 0.02, 0.15, and 0.35 are interpreted as small, medium, and large effects, respectively. These findings indicate that the interaction between PE scheduling parameters and demographic factors contributes meaningfully, though not strongly, to variations in predicted fitness outcomes.

## 5 Discussion and recommendations

PE plays a vital role in the promotion of long-term health benefits, but its optimization has often been overlooked in academic research and policy making. This study introduces an efficient method that uses DL to optimize PE schedules. It addresses key gaps in understanding the relationship between PE activities and fitness outcomes. The findings demonstrate that data-driven approaches can significantly enhance the effectiveness of PE programs.

### 5.1 Discussion

The results demonstrate that the proposed deep learning model significantly outperforms existing state-of-the-art approaches in predicting fitness score improvements. This is reflected in key performance metrics, including a 1.35% reduction in MSE, a 1.22% improvement in MAE, and a 1.18% increase in *R*^2^. These enhancements underscore the model's robustness in capturing intricate relationships among PE schedules, demographic attributes, and physical health indicators.

Beyond predictive accuracy, the study emphasizes the value of integrating varied PE activities, appropriate session durations, and ongoing evaluations into school programs. The model's architecture, particularly the fusion layer enables the combined analysis of spatial and temporal features, resulting in precise, data-driven recommendations. The consistency of performance across multiple demographic segments further validates the model's adaptability in diverse educational settings.

Moreover, the findings suggest potential health benefits extending beyond immediate fitness improvements. Specifically, the model may contribute to mitigating risk factors associated with metabolic syndrome (MetS), which includes central obesity, elevated blood pressure, and impaired glucose regulation. Prior studies have linked insufficient physical activity to the progression of MetS in children and adolescents (66). By optimizing PE schedules to promote regular, effective activity, the proposed model may help reduce early markers of MetS and support long-term cardiometabolic health.

### 5.2 Synthetic data alignment and model accuracy

The use of synthetic data generation ensured that the model could adapt to real-world variations in physical activity patterns, health trends, and PE participation rates. Real-world datasets often exhibit seasonal changes, behavioral inconsistencies, and external influences (e.g., weather, school policies, and accessibility to facilities), which can affect PE participation and fitness outcomes. By introducing synthetic variations, the model was exposed to a broader spectrum of potentially unseen conditions, enhancing its generalizability.

The integration of synthetic PE participation scenarios enabled the model to better generalize to diverse student populations. For instance, by generating variations in PA intensity levels and session durations, the model learned to predict fitness improvements under different constraints, such as limited PE access in rural schools vs. structured fitness programs in urban institutions. Additionally, temporal noise injection simulated daily variations in student engagement levels, ensuring that the model did not overfit to idealized, static patterns.

To evaluate the impact of synthetic data on model accuracy, comparative analyses were conducted between models trained solely on real-world data and those trained on a combination of real and synthetic data. The results demonstrated a reduction in prediction errors, with an improvement of 2.8% in MSE and a 3.1% reduction in MAE. These improvements indicate that incorporating synthetic data enhanced the model's robustness by preventing it from being biased toward specific demographic or environmental conditions.

### 5.3 Recommendations

Based on the findings, several recommendations are proposed to maximize the effectiveness of PE schedules. Table 2 shows the optimization recommendations for the PE schedule designed to maximize long-term health benefits. It provides detailed insights into various strategies, their target audience, and the expected outcomes. These recommendations aim to guide educators, policymakers, and communities in the implementation of effective PA programs that promote fitness and wellbeing. By using data-driven approaches, the proposed model ensures equitable access and sustainable improvements in student health outcomes.

**Table 2:** Extended PE schedule optimization recommendations.

**Recommendation**	**Details**	**Audience**	**Expected outcome**
Personalized PE schedules	Design schedules based on individual fitness levels, age, and health goals.	Students and educators	Improved fitness levels tailored to individual needs.
Incorporate varied activities	Include a mix of aerobic, strength, and flexibility activities to ensure comprehensive development.	Students and educators	Comprehensive physical development and student engagement.
Optimize session durations and frequency	Structure consistent sessions (30–60 min) multiple times per week for effectiveness.	Students and educators	Enhanced effectiveness of PE sessions with optimal scheduling.
Monitor fitness progress	Track fitness score improvements periodically to adjust schedules dynamically.	Educators and administrators	Dynamic adjustments to PE schedules based on real-time data.
Leverage data-driven insights	Use predictive analytics to identify effective PE combinations for maximum benefits.	Policymakers and administrators	Data-backed strategies for maximum health benefits.
Encourage active breaks	Incorporate shorter active breaks during the day to reduce sedentary behavior.	Students and educators	Reduced sedentary behavior and improved overall activity levels.
Training for educators	Train teachers and coaches to implement data-driven PE strategies effectively.	Educators and trainers	Efficient implementation of data-driven strategies in schools.
Engage families and communities	Encourage families and communities to support active lifestyles through initiatives.	Families and communities	Support for active lifestyles beyond school settings.
Promote equitable access	Ensure all students have access to structured PE programs, adapting as needed.	Policymakers and educators	Equitable access to health-promoting PE programs.
Evaluate and iterate	Regularly evaluate health metrics and use feedback to refine programs.	Educators and administrators	Sustained program effectiveness through regular refinement.

While these recommendations provide a strong framework for optimizing PE schedules, their implementation in resource-limited settings and schools with diverse demographics requires adaptability and innovation. Below are examples of how these strategies can be applied in low-resource schools and diverse student populations.

(a) **Personalized PE schedules with minimal resources:** in schools with limited access to gym equipment, personalized PE schedules can be adapted by utilizing bodyweight exercises (e.g., push-ups, squats, jumping jacks) and outdoor activities like running or circuit training in open fields. Teachers can use simple fitness assessment tools (such as timed runs or endurance tests) to categorize students into low, moderate, and high-intensity groups without the need for advanced tracking devices.
(b) **Incorporate varied activities in multi-cultural and mixed-ability settings:** schools with diverse student populations can integrate culturally inclusive physical activities such as traditional dance, martial arts, or regional sports to engage students from different backgrounds. Additionally, adaptive physical education techniques (e.g., seated exercises, inclusive team sports) can be incorporated to accommodate students with disabilities.
(c) **Data-driven insights with basic tools:** even in the absence of high-tech fitness trackers, teachers can manually record student participation, endurance levels, and progress over time in simple spreadsheets or paper-based logs. Schools can implement low-cost digital solutions, such as free mobile apps for tracking physical activity trends.
(d) **Encourage active breaks without disrupting curriculum:** in schools with rigid academic schedules, short movement breaks (e.g., 5-min stretching, classroom-based exercises) can be introduced between lessons to reduce sedentary behavior without requiring additional resources. For example, schools in crowded urban areas with limited playgrounds can incorporate standing desks, hallway stretching routines, or stair exercises to increase movement.
(e) **Engage communities to overcome resource barriers:** in economically disadvantaged areas where schools lack sports facilities, community partnerships can be established with local parks, recreational centers, or non-profit organizations to provide after-school PE programs. Schools can also leverage volunteers, parents, and local coaches to assist in delivering structured physical activities.
(f) **Equitable access through policy adjustments:** to ensure that students in rural and low-income schools receive adequate PE, policymakers can advocate for flexible scheduling, allowing for rotational sports programs where different age groups use shared equipment on different days. Additionally, partnerships with public health initiatives can help provide free or subsidized sports gear to underprivileged students.
(g) **Feedback mechanisms for dynamic adjustments:** to further enhance PE scheduling, schools can introduce student feedback loops where teachers collect data on energy levels, engagement, and activity preferences after each session. This information can be used to adjust future PE sessions dynamically. In low-resource settings, simple tools such as weekly student reflection logs or teacher-led discussions can serve as effective feedback mechanisms. In technology-equipped schools, mobile surveys or AI-driven adaptive schedules could be employed to refine PE programs in real time.

### 5.4 Implications for public health

The proposed framework has significant implications for public health. Its scalability and adaptability make it a valuable tool for designing PE programs that promote fitness and well-being. Key implications are summarized as follows.

(a) **Enhanced individual health outcomes:** by addressing the diverse fitness needs of students, the proposed model ensures improved physical fitness and overall wellbeing for individuals.
(b) **Broader societal benefits:** effective PE programs can lead to societal advantages such as reduced healthcare costs, improved quality of life, and increased productivity.
(c) **Promotion of data-driven strategies:** the integration of predictive analytics allows policymakers and educators to base decisions on evidence, ensuring the effectiveness of interventions.
(d) **Sustainability in health improvements:** the proposed model supports long-term health benefits by fostering active lifestyles that continue beyond the classroom.
(e) **Equitable access to health resources:** the approach ensures that PE programs cater to all students, including those from underserved communities or with physical limitations.
(f) **Alignment with public health goals:** by focusing on preventive measures, the framework contributes to national and global health objectives aimed at reducing the burden of chronic diseases.

### 5.5 Limitations and future directions

While the proposed model demonstrates strong predictive performance, it still suffers from several limitations.

- First, the dataset used for model training, though comprehensive, may not fully represent diverse global populations. The study primarily focuses on data from structured PE programs, which may not generalize to regions with different educational policies, cultural attitudes toward physical activity, or resource constraints. Future research should extend datasets to include underrepresented populations, cultural variables, and environmental factors to improve model robustness and generalizability.
- Second, the current model does not explicitly account for socio-cultural influences on physical activity engagement. Factors such as gender norms, traditional exercise practices, and regional accessibility to sports facilities can significantly impact student participation in PE. Future studies should integrate cultural and behavioral variables into the model to enhance its adaptability across different demographic groups (5).
- Third, while the model optimizes PE schedules based on historical fitness data, it does not incorporate real-time adaptive mechanisms. A promising direction for future research is the development of dynamic feedback loops that adjust PE schedules based on student engagement levels, real-time performance tracking, and wearable fitness data. Reinforcement learning approaches could be used to ensure continuous optimization of physical activity plans (42, 43).
- Fourth, the reliance on structured PE programs may limit applicability in informal or home-based exercise settings. As hybrid and remote learning environments continue to evolve, future work could explore home-based PE interventions, using mobile applications and virtual coaching to provide adaptive exercise recommendations personalized to individual student needs.

Despite these limitations, this study establishes a robust framework for evidence-based PE scheduling, offering actionable insights for educators, policymakers, and researchers. The proposed DL-based scheduling model can be deployed as a decision-support tool in school systems and health departments to guide the planning of adaptive PE programs. It could be integrated into school health dashboards, community fitness initiatives, or mobile platforms to offer real-time activity recommendations, fitness tracking, and personalized scheduling strategies. Policymakers could also utilize the model to inform policy adjustments aimed at reducing disparities in physical activity access and outcomes among students from diverse backgrounds.

## 6 Conclusion

This study introduced a DL model that optimized PE schedules to maximize long-term health benefits. The proposed model accurately predicted improvements in fitness scores. Therefore, the proposed model can effectively capture complex relationships between PE schedules and health outcomes. Compared to state-of-the-art models, the proposed approach achieved significant improvements in predictive accuracy. This was evidenced by reductions in MSE and MAE and higher *R*^2^ values by 1.35%, 1.18%, and 1.22%, respectively. The findings underscored the importance of data-driven approaches in designing PE schedules that met the diverse needs of the students. This ultimately promoted healthier and more active lifestyles.

## Data Availability

The original contributions presented in the study are included in the article/Supplementary material, further inquiries can be directed to the corresponding author.
